# Recent Advances in Excimer-Based Fluorescence Probes for Biological Applications

**DOI:** 10.3390/molecules27238628

**Published:** 2022-12-06

**Authors:** Yi Chen

**Affiliations:** 1 Key Laboratory of Photochemical Conversion and Optoelectronic Materials, TIPC, CAS, Beijing 100190, China; yichen@mail.ipc.ac.cn; 2 University of Chinese Academy of Sciences, Beijing 100190, China

**Keywords:** fluorescent probe, excimer, biological application

## Abstract

The fluorescent probe is a powerful tool for biological sensing and optical imaging, which can directly display analytes at the molecular level. It provides not only direct visualization of biological structures and processes, but also the capability of drug delivery systems regarding the target therapy. Conventional fluorescent probes are mainly based on monomer emission which has two distinguishing shortcomings in practice: small Stokes shifts and short lifetimes. Compared with monomer-based emission, excimer-based fluorescent probes have large Stokes shifts and long lifetimes which benefit biological applications. Recent progress in excimer-based fluorescent sensors (organic small molecules only) for biological applications are highlighted in this review, including materials and mechanisms as well as their representative applications. The progress suggests that excimer-based fluorescent probes have advantages and potential for bioanalytical applications.

## 1. Introduction

Excimer is an excited state dimer produced by molecular photo-association. Excimers are classified into two different types: one is dynamic excimers, the other static excimers [1]. The former is produced from the collision between the excited-state and ground-state molecules, the latter obtained upon photoexcitation of a ground-state dimer generated by weak molecular interactions such as π–π stacking or H-bond. Excimers can also be divided into two categories: intermolecular excimer and intramolecular excimer [2]. The formation of excimers and their fluorescence can be modulated by the concentration of corresponding monomers and external stimuli such as pH, environmental temperature, solvent polarity and chemical species [3,4,5,6]. The exceptional sensitivity of excimers could be used to monitor fine conformation within molecules, which is particularly useful in bioanalysis of nucleic acids [7,8,9,10] and proteins [11,12,13,14].

Excimer-based fluorescent probes have many advantages over conventional monomer-based fluorescent probes. On the one hand, excimer-based fluorescent probes yield specific fluorescence emissions at much longer wavelengths than the corresponding monomer emissions. Consequently, large Stokes shifts are obtained, which can effectively avoid fluorescence self-absorption. On the other hand, excimer-based fluorescent probes exhibit longer fluorescent lifetimes than these of monomers, which benefit measurements especially when the auto-fluorescence of biological species is high. Furthermore, excimer-based fluorescent probes can display dual emissions which can be used for ratiometric signaling. In various strategies for constructing ratiometric probes [15,16,17,18], an elegant approach is to modulate the monomer and its excimer emission with a single fluorophore molecule. Therefore, researchers have made every effort to find excimer-based fluorescent probes for various purposes such as the detection of essential elements [19,20,21,22,23], chemical warfare agents and explosives [24,25,26] and biomolecules [27,28,29,30]. This review is a collection of recent research articles discussed in the context of excimer-based fluorescent probes organized according to their fluorescent core scaffold and with their representative biological applications.

## 2. Design Strategies for Excimer-Based Fluorescent Probes

**Figure 1:**
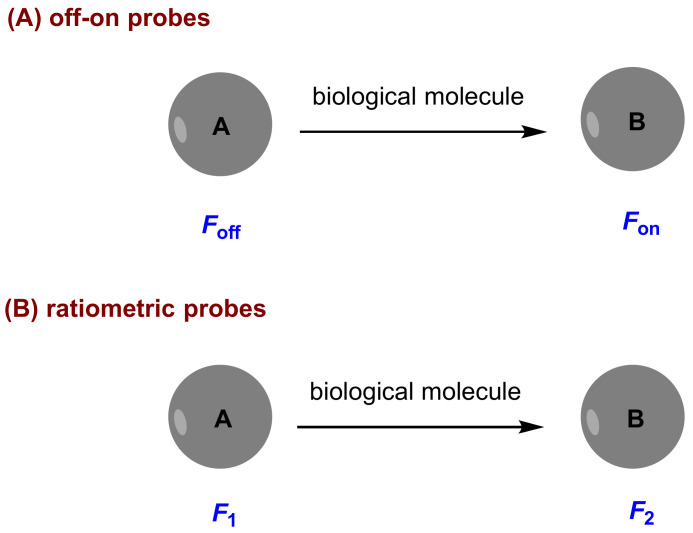
Schematic representation of fluorescent off-on probes and ratiometric probes in this context.

A valuable fluorescent probe for biological application should have some excellent qualities including high selectivity, high sensitivity, accurate quantitative analysis and facile preparation. To this end, probes with some characteristics including “off-on” fluorescence, red or near-infrared (NIR) fluorescence and ratiometric measurement are desired. Off-on fluorescence exhibits low background signal as compared to on-off fluorescence, and provides high detection sensitivity. Red and NIR fluorescence supplies less light scattering and minimized tissue auto-fluorescence background. Ratiometric measurement can provide reliable quantitative analysis because of built-in self-calibration for signal correction by measuring the ratio of the intensity profiles at different wavelengths. In this review, two kinds of fluorescent probes are involved: one is off-on probes, and the other ratiometric probes (Figure 1).

## 3. Fluorescent Probes and Their Biological Applications

### 3.1. Pyrene-Based Probes

Pyrene is an aromatic compound with a strong emission in solution. The aggregation of monomers (λ_em_ = 370–420 nm) can produce excimer (λ_em_ = 450–550 nm) [31], and monomers can be restored through deaggregation [32]. Pyrene excimer emission provides large Stokes shifts (ca. 140 nm) and long fluorescence lifetimes (40–60 ns). These particular properties have been used for the design of many fluorescent probes.

**Figure 2:**
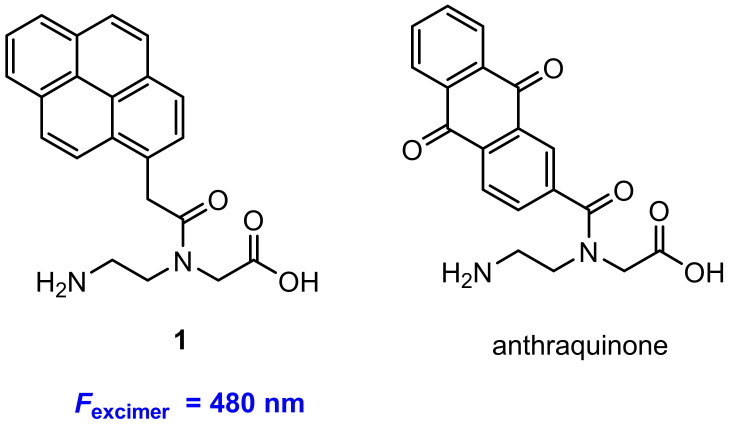
Chemical structures of fluorophore **1** and quencher anthraquinone.

**Figure 3:**
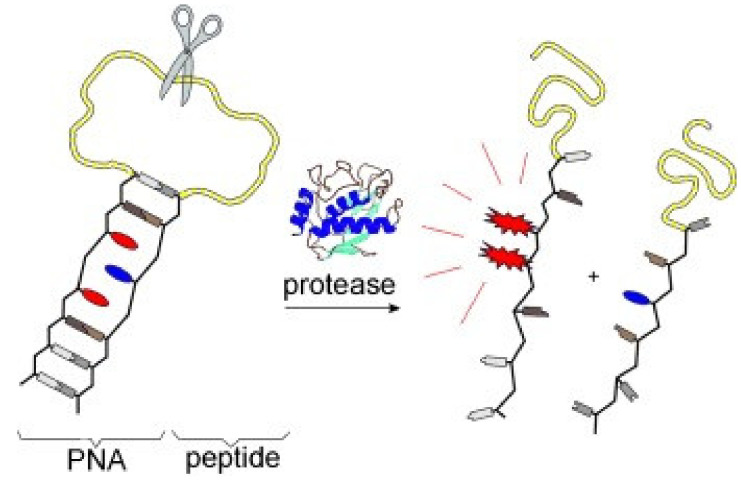
Schematic representation for the detection of protease (fluorophore **1**, red color in PNA; quencher anthraquinone, blue color in PNA) [33].

Fischbach and co-workers [33] developed a class of pyrene excimer-based fluorescent probes for the detection of protease. They employed a pyrene derivative (**1**) and anthraquinone (Figure 2) as fluorophore and quencher respectively. A peptide segment serving as the protease substrate was equipped with self-complementary peptide nucleic acid (PNA) arms. The formed PNA duplex results in a hairpin-type arrangement, in which both fluorophore and quencher are forced into proximity. The probe mechanism is that the pyrene excimer fluorescence is restored upon protease-catalyzed scission of a peptide substrate (Figure 3). With incubation with protease MMP-7 [34], a 48-fold enhancement of fluorescence at 480 nm was obtained.

**Figure 4:**
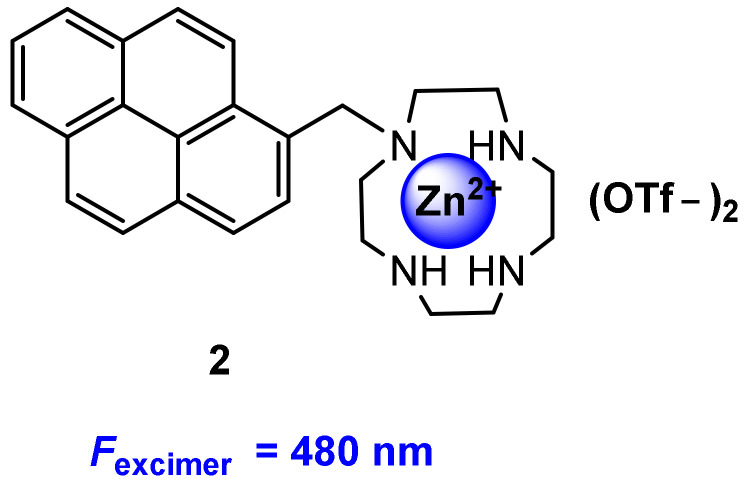
Chemical structure of probe **2**.

**Figure 5:**
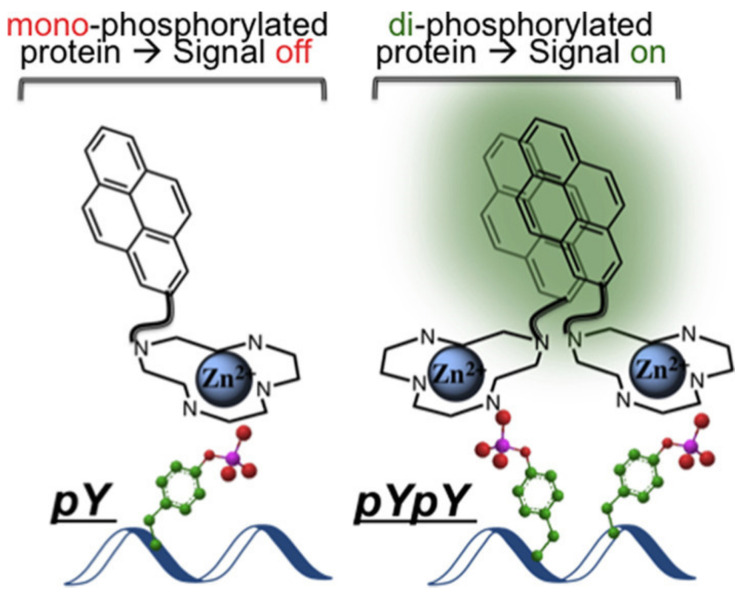
Schematic representation for the detection of diphosphorylated protein sites [35].

Kraskouskaya and co-workers [35] reported a pyrene excimer-based fluorescent probe for the selective detection of proximally diphosphorylated protein sites. Compound **2** used as probe (Figure 4) was synthesized by coupling a pyrene to Zn(II)-cyclen macrocycle. The probe mechanism is as follows: Diphosphorylation on proximal residues is required for the activation of a subset of proteins, resulting in pYpY (pY: staining all tyrosine residues) and pTXpY motifs (pT: staining all threonine residues), respectively (X = any amino acid). The probe formed a 1:1 complex with a pX-containing peptide/protein site, and therefore no excimer signal. However, for proximally diphosphorylated peptides, each pX residue can coordinate a Zn(II)-cyclen unit, promoting pyrene interaction and resulting in excimer emission (Figure 5).

**Figure 6:**
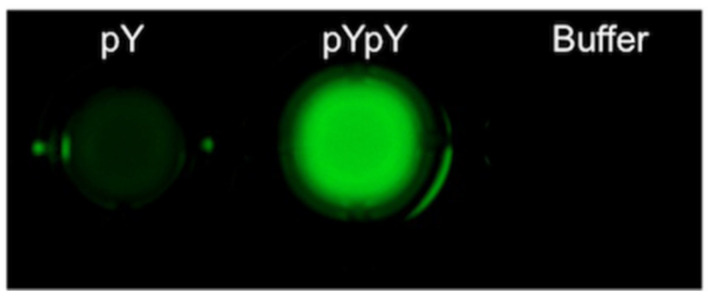
Fluorescence image of peptides (25 μM) by treatment with probe **2** (50 μM) under Bio-Rad ChemiDoc MP imaging system [35].

The evaluation of probe **2** was performed in HEPES using mono- and diphosphorylated sites (AYpYAA and ApYpYAA) as models. With 350 nm excitation, the probe produced a distinct shift of fluorescence from 380 to 480 nm in response to diphosphorylated peptides. The intensity of fluorescence at 480 nm was much larger than that of the response to monophosphorylated pY peptides, which suggested that the strong excimer fluorescence is specific to a diphosphorylated motif. Furthermore, titration experiments showed that the maximum excimer signal was obtained from a **2**-peptide (2:1) complexation, in which the detection limit is calculated to be 0.6 μM. In addition, the selective response by fluorescence imaging was also observed (Figure 6), which may broaden the use of the probe.

**Figure 7:**
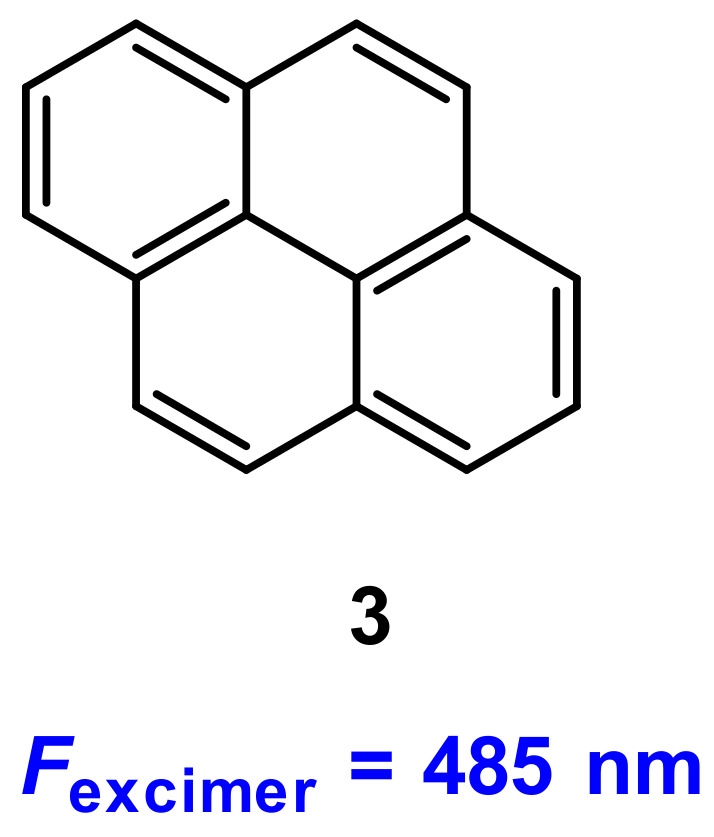
Chemical structure of probe **3**.

**Figure 8:**
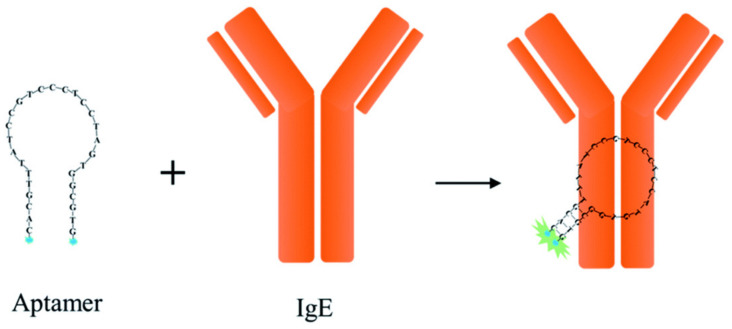
Schematic representation for the detection of IgE [36].

**Figure 9:**
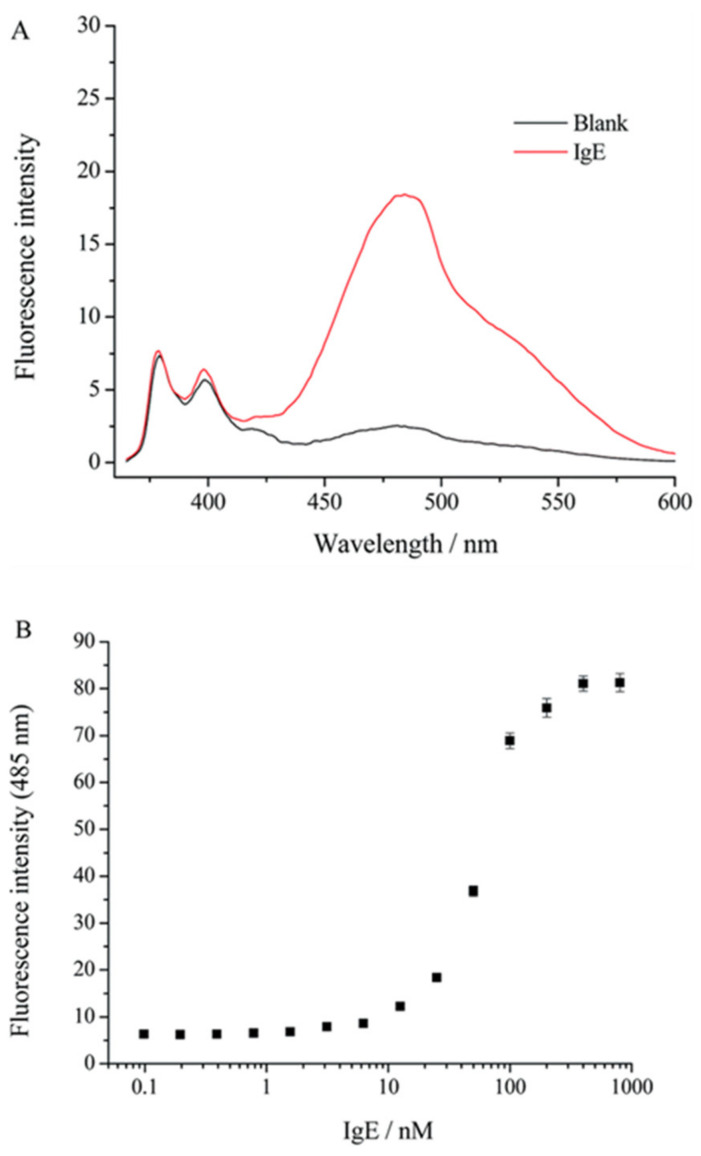
(**A**) The fluorescence spectra of the probe **IgE-4bp-2Py** (50 nM) in the absence and the presence of IgE (50 nM). (**B**) The fluorescence intensity (485 nm) at different concentrations of IgE [36].

Human immunoglobulin E (lgE) plays a pivotal role in various allergic reactions. Bai and co-workers [36] used pyrene (**3**) as fluorophore (Figure 7) for lgE sensing. A aptamer-based probe was constructed with four base pairs at the stem to which pyrene was labeled at the 3′ and 5′ end, respectively (Figure 8). Upon binding to lgE, the pyrene moiety at each terminal of the aptamer came into proximity, creating an excimer with emission at 485 nm (Figure 9). Under optimized conditions, the detection limit for lgE is calculated to be 1.6 nM.

**Figure 10:**
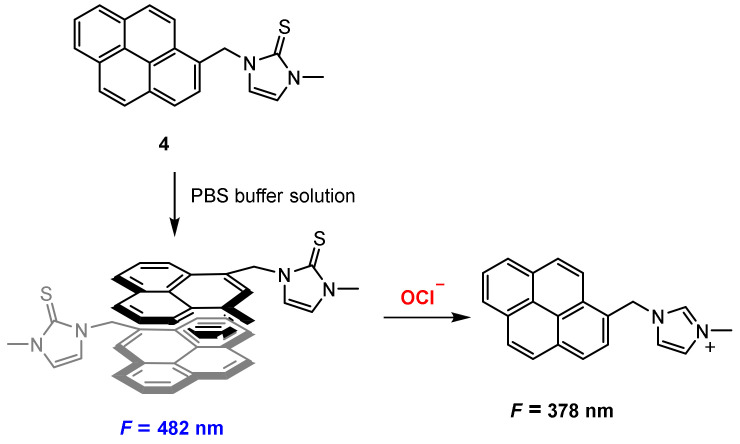
Chemical structure of probe **4** and its excimer response to OCl^−^.

Pak and co-workers [37] developed a chemical reaction-based ratiometric fluorescent probe **4** (Figure 10) for detection of hypochlorite (OCl^−^). Probe **4** was constructed by the conjugation of a pyrene unit as fluorophore and an imidazoline-2-thione as a reactive site. The probe aggregated to excimer in PBS (pH = 7.4) with fluorescence at 480 nm, and upon reaction with OCl^−^, the reactive site imidazoline-2-thione was oxidized to imidazoline, which exhibited monomer fluorescence at 378 nm.

In PBS buffer solution (pH 7.4), probe **4** exhibited a strong excimer fluorescence at 482 nm. The addition of OCl^−^ to the solution of probe **4** resulted in a decrease of the fluorescence at 482 nm and appearance of monomer fluorescence at 378 nm. The ratio of the fluorescence intensities (*F*_482_/*F*_378_) was reduced 124-fold when OCl^−^ (0–40 μM) was added. The probe exhibited good sensitivity and the detection limit is calculated to be 0.2 μM.

**Figure 11:**
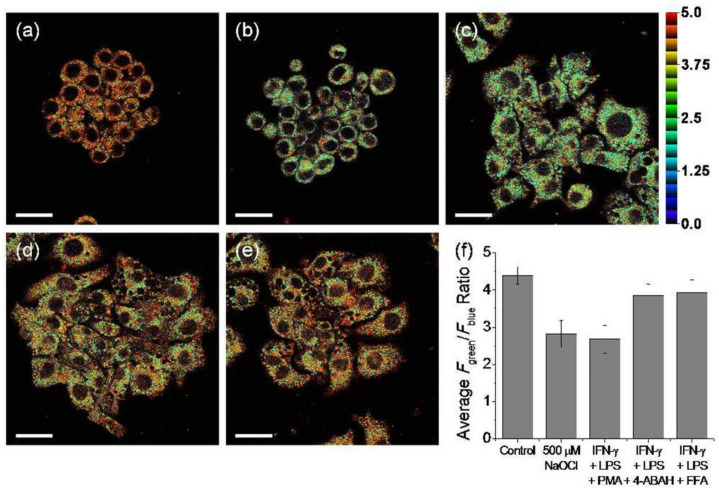
Pseudocolored ratiometric two-photon microscopic images of Raw 264.7 cells incubated with probe **4** (5 μM) for 30 min. Cells were pretreated with (**a**) 0; (**b**) NaOCl (500 μM, 30 min); (**c**) LPS (100 ng/mL, 16 h), IFN-γ (50 ng/mL, 4 h, PMA (10 nM, 30 min); (**d**) LPS, IFN-γ, PMA, and 4-ABAH (50 μM, 4 h); and (**e**) LPS, IFN-γ, PMA, and FFA (50 μM, 4 h) before treated with **7**. (**f**) Average *F*_green_/*F*_blue_ ratios of the corresponding two-photon images. 730 nm excitation and 380–430 nm (blue) and 480–600 nm (green) emission windows. Scale bars = 25 μm [37].

Ratiometric tracking of endogenous OCl^−^ was conducted in stained Raw 264.7 live cells with probe **4** using two-photon excitation. Endogenous OCl^−^ was produced with ROS inducers (LPS: 100 ng/mL, IFN-γ: 50 ng/mL, and PMA: 10 nM). Upon excitation with 730 nm, the average fluorescence intensity of the stained cells was reduced, and the ratio of *F*_green_/*F*_blue_ is calculated to be 2.7 (Figure 11c,f). However, the ratio of *F*_green_/*F*_blue_ increased to 3.9 when inhibitors such as 4-aminobenzoic acid hydrazide (4-ABAH) [38] or flufenamic acid (FFA) [39] were added (Figure 11d–f). The results suggest that **7** could be applicable to ratiometric fluorescence imaging of OCl^−^ in live cells.

**Figure 12:**
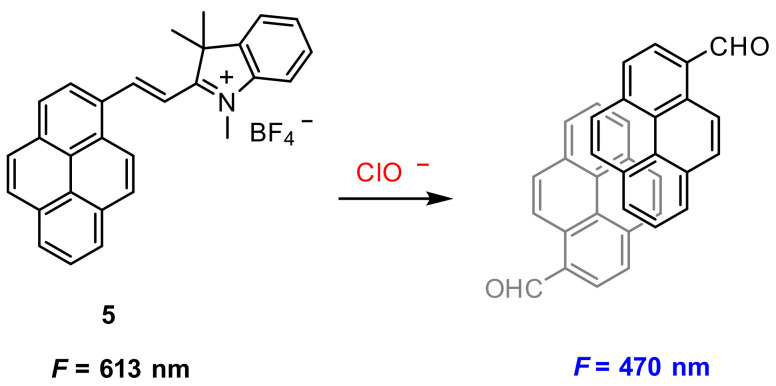
Chemical structure of probe **5** and its excimer response to OCl^−^.

Wu and co-workers [40] also reported a chemical reaction-based ratiometric fluorescent probe for the detection of OCl^−^. Probe **5** was constructed by the conjugation of pyrene and 1,2,3,3-Tetramethyl-3H-indolium. The double bond between pyrene unit and indolium moiety in probe **5** can be broken by OCl^−^ to generate pyrenecarboxaldehyde, the latter forming excimer in PBS (pH 7.4) (Figure 12). This process correspondingly led to fluorescence changes from 613 nm to 470 nm and thus affords the ratiometric fluorescent detection of OCl^−^. The detection limit is determined to be 0.35 μM.

**Figure 13:**
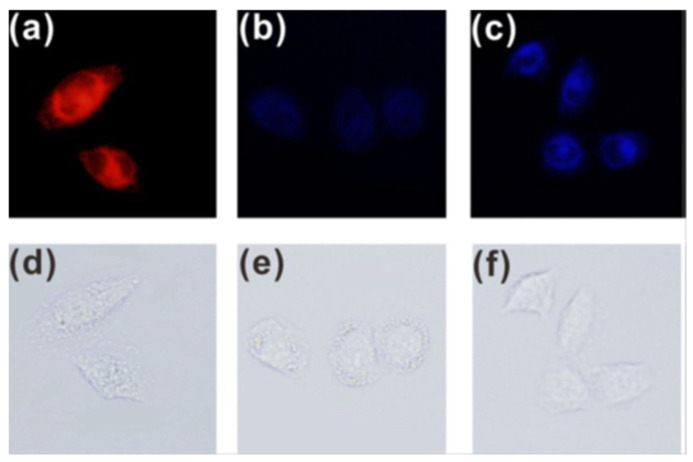
Fluorescence images of Hela cells incubated with probe **5** (10 μM) before (**a**,**d**) and after being treated with ClO^−^ (10 μM) (**b**,**e**) and ClO^−^ (50 μM) (**c**,**f**) for 2 h [40].

Bioimaging of OCl^−^ in live cells was performed in stained HeLa and Raw 264.7, respectively. Upon ClO^−^ loading and incubation for 2 h, blue fluorescence signal was observed, suggesting the internalization of **5** and its reaction with the preloaded ClO^−^ (Figure 13).

**Figure 14:**
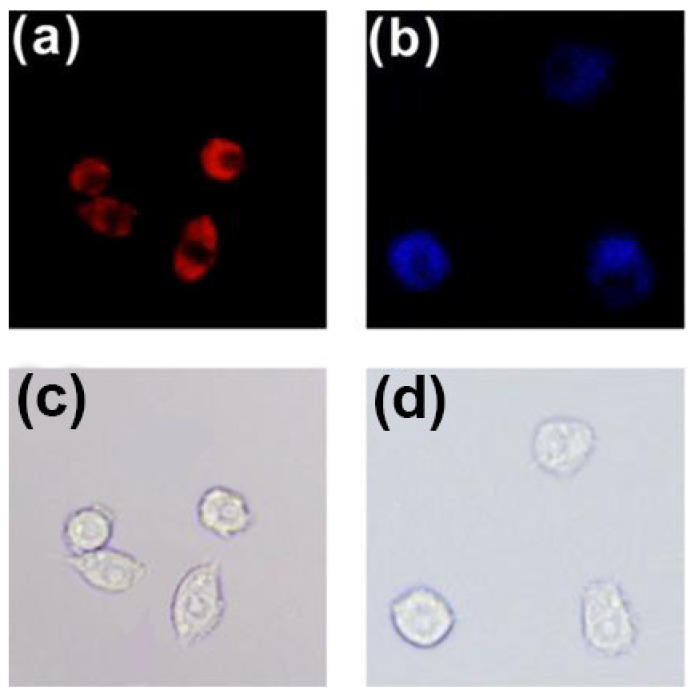
Fluorescence images of endogenous ClO^−^ in RAW 264.7 cells. (**a**,**c**) The cells incubated with probe **5** (10 μM) for 2 h. (**b**,**d**) The cells treated with PMA (2.0 mg/mL) for 30 min and then incubated with probe **5** (10 μM) for 2 h [40].

Endogenous ClO^−^ in RAW 264.7 live cells was obtained by PMA (phorbol myristate acetate) [41]. As shown in Figure 14, red fluorescence was observed when the RAW 264.7 cells were incubated only with probe **5**. However, when incubated with 2 mg/mL PMA and **5**, the cells displayed blue fluorescence.

**Figure 15:**
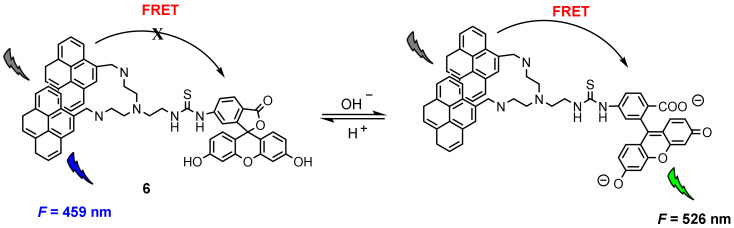
Chemical structure of probe **6** and its response to pH.

Fluorescence resonance energy transfer (FRET) is a nonradiative process and occurs when the excited-state energy of the donor fluorophore is transferred to the acceptor fluorophore in close proximity (≤10 nm) through long-range dipole–dipole interactions [42,43]. Wu and co-workers [44] constructed a FRET-based fluorescent probe for ratiometric quantitative monitoring of pH changes in living cells. Probe **6** was composed of two pyrene units and one fluorescein moiety conjugated with a linker (Figure 15), in which a bispyrene moiety served as the energy donor and fluorescein as the energy acceptor. The fluorescence at 526 nm (*F*_526_) increased upon increasing pH values, while the fluorescence at 459 nm (*F*_459_) decreased concomitantly; the relative ratio of *F*_526_/*F*_459_ increased from 0.26 to 5.82 over the pH range of 3.0–10.0.

**Figure 16:**
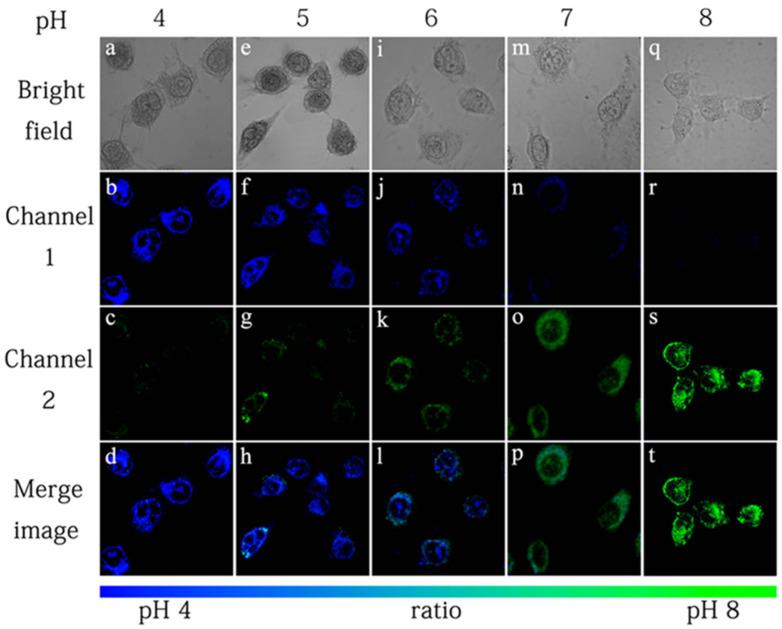
Fluorescence images of probe **6** (10.0 μM) in HeLa cells with pH 4 (**a**–**d**), pH 5 (**e**–**h**), pH 6 (**i**–**l**), pH 7 (**m**–**p**) and pH 8 (**q**–**t**). Channel 1 was collected at 440–480 nm (pyrenyl excimer). Channel 2 was collected at 510–550 nm (fluorescein) [44].

The applicability of probe **6** to quantifying intracellular pH was carried out in HeLa cells with H^+^/K^+^ ionophore nigericin [45]. The intensity of fluorescence from pyrenyl excimer (Figure 16, Channel 1) decreased, whereas the fluorescence from fluorescein (Figure 16, Channel 2) increased with the increase of the pH value. It is worth noting that the color of cell imaging displayed a significant change from blue to green when intracellular pH was changed from 6 to 7, which may satisfy the requirement for real-time observations. The relative ratio of intracellular fluorescence intensities (*F*_channel2_/*F*_channel1_) increased from 0.27 to 2.25 when the pH value increased from 4.0 to 8.0 in HeLa cells.

### 3.2. Perylene-Based Probes

Perylene and its derivatives have attracted significant interest due to their outstanding optical and electronic properties [46,47,48]. They have low LUMO orbitals, prominent photo-/thermal stability and high fluorescence quantum yields. In addition, the π-stacked perylene derivatives have strong electronic communications among the individual chromophores in close proximity in both their ground and excited states [49,50], resulting in excimers/aggregates with distinct red fluorescence [51,52,53]. A growing number of reports have utilized perylene derivatives as fluorescence probes for tracking, biolabeling, base-mismatch detection, biomolecule sensing and bioimaging [54,55,56,57,58].

**Figure 17:**
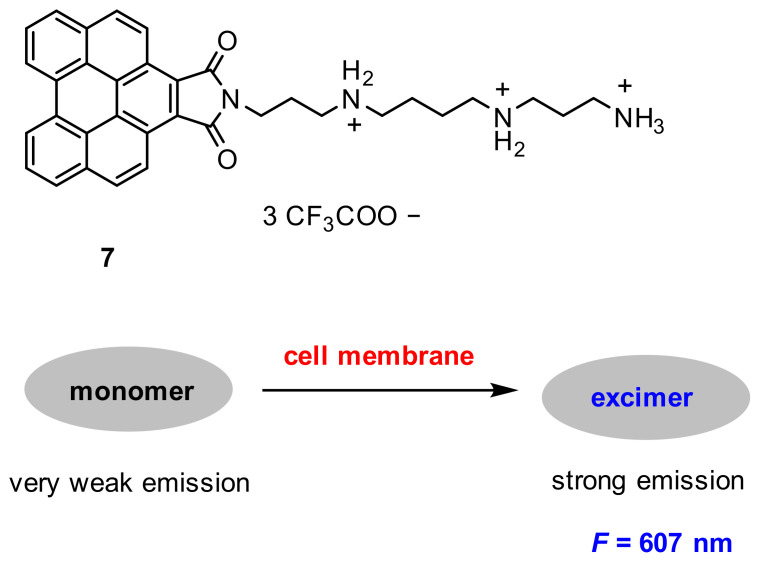
Chemical structure of probe **7** and its response to cell membrane.

Niu and co-workers [59] developed a turn-on benzoperylene excimer-based probe for wash-free cell membrane fluorescence imaging. Probe **7** was constructed with a hydrophilic spermine chain and a hydrophobic benzoperylene fluorophore group (Figure 17). Probe **7** showed a very weak emission in monomer, but strong emission (λ = 607 nm) in excimer, and the excimer emission exhibited sensitivity to the micro-environment.

**Figure 18:**
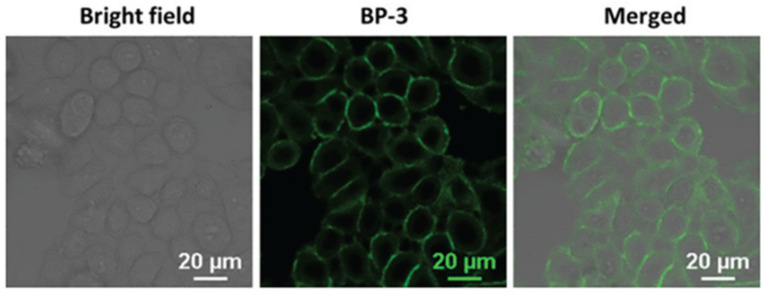
Fluorescence images of MCF-7 cells stained with probe **7** (10 μM). Scale bar: 20 μm [59].

After MCF-7 live cells were incubated with probe **7** (10 µM) for 5 min, a remarkable fluorescence in the cell membrane was observed (Figure 18). The fluorescence was derived from the aggregation of probe **7** in the membrane due to both electrostatic and hydrophobic interactions between the probe and the lipid membrane. Furthermore, probe **7** exhibited some advantages of excellent solubility, good selectivity and large Stokes shift.

**Figure 19:**
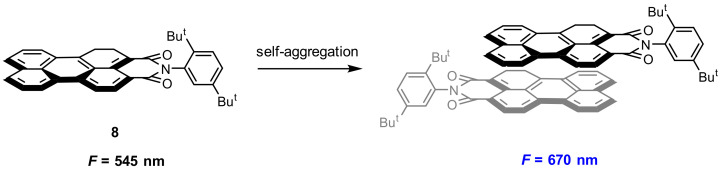
Chemical structure of probe **8** and its self-aggregation.

Molecular self-assembly is an important approach in the construction of functional materials for biological application. Perylene and its derivatives exhibit distinct fluorescence in solution and in the aggregation state, and the fluorescence can be fine-tuned by exoteric factors [60,61,62]. Jana and co-workers [63] reported a new strategy for enhancement of excimeric NIR fluorescence by morphology tuning of self-assembled perylene derivative from nanoparticles to colloidosomes. Perylene derivative **8** (Figure 19) was synthesized and used as fluorescent dye, which displayed entirely distinct fluorescence in a different morphology by self-aggregation. Probe **8** showed a green emission (545 nm) in a monomer state and red emission (670 nm) in the aggregation (excimer). With a biocompatible nonionic surfactant pluronic F127, two micelles **MM-8@plu127** (emission at 575 nm) and **EM-8@Plu127** (emission at 670 nm) were obtained.

**Figure 20:**
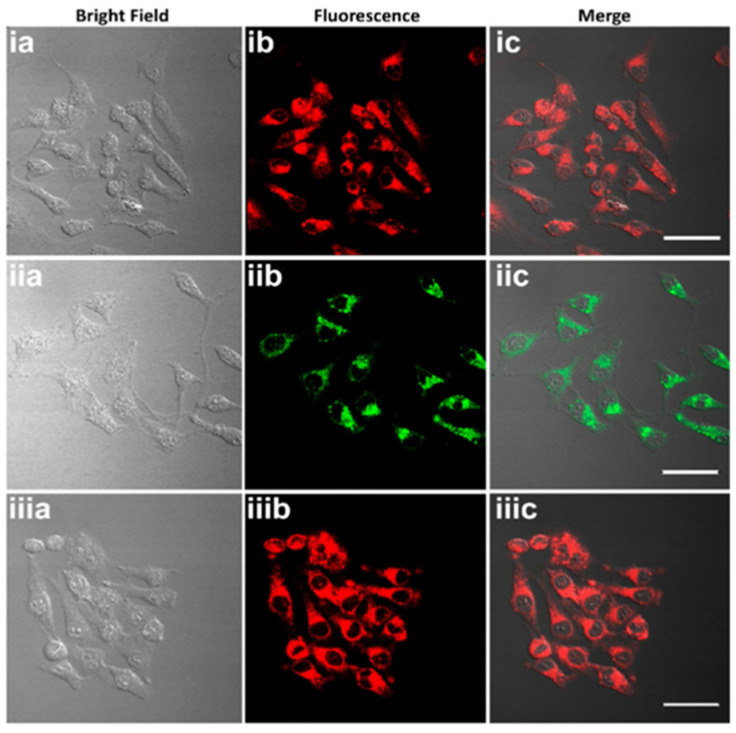
(**a**) Bright-field, (**b**) fluorescence, and (**c**) merged images of HeLa cells incubated with (**i**) probe **8** NPs in water, (**ii**) **MM-8@ Plu127**, and (**iii**) **EM-8@Plu127**, scale bar: 50 μm. Emission was recorded using 550 nm channel for the green emission and 700 nm channel for the NIR emission [63].

The bioimages of probe **8** in different morphology (**8** NPs, **MM-8@plu127**, and **EM-8@Plu127**) were performed in HeLa cells. Cellular uptake after 4 h of incubation revealed that all the three types were readily internalized by the HeLa cells (Figure 20) and were mainly localized in the cytoplasm.

**Figure 21:**
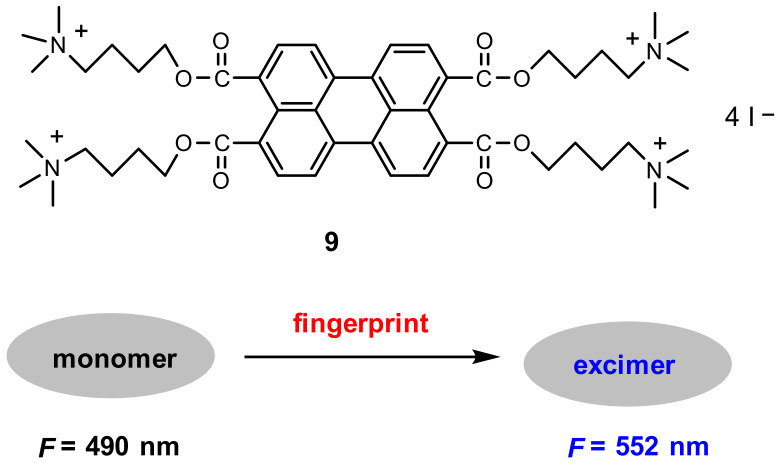
Chemical structure of probe **9** and schematic representation for the detection of fingerprint.

As a powerful tool to validate identity, fingerprint identification has been used in a variety of fields including access control, forensic investigation and medical diagnostics [64,65]. Based on perylene excimer fluorescence, Wang and co-workers [66] developed a probe for on-site detection of latent fingerprints. Probe **9** is composed of one perylene core and four positively charged quaternary ammonium groups (Figure 21).

**Figure 22:**
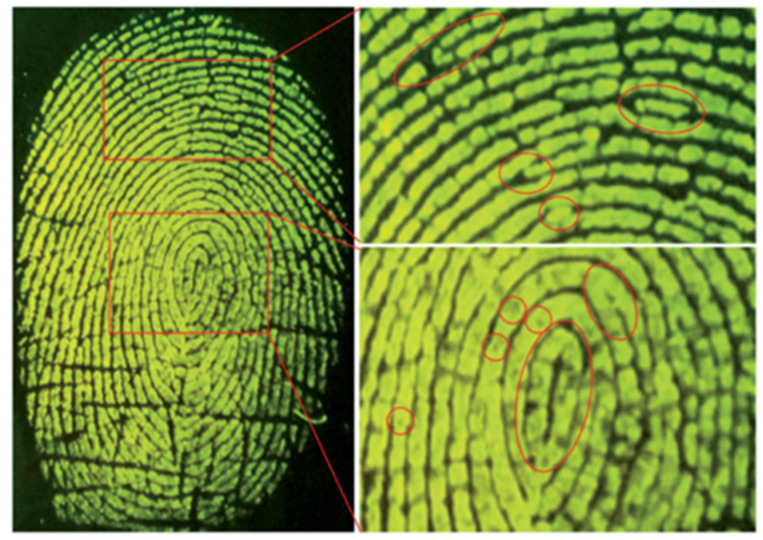
Fluorescence image of fingerprint on a PVDF membrane with a bandpass filter [66].

The fluorescence image of the latent fingerprint was observed when a substrate containing a latent fingerprint was submerged into the developer solution (0.2 mM **9** in 20 mM Tris–HCl buffer, pH 7.4) upon excitation with 365 nm light (Figure 22). Study of the mechanism showed that probe **9** displayed monomer fluorescence at a low concentration but excimer fluorescence at a high concentration. The fluorescence in the latent fingerprint resulted from the excimer of probe **9** due to the aggregation induced by electrostatic and hydrophobic interactions between the probe and fatty acids and other organic components in the fingerprint residues. The control experiment confirmed that no developed fingerprint fluorescence image was observed when a negatively charged perylene probe was used instead of probe **9**.

**Figure 23:**
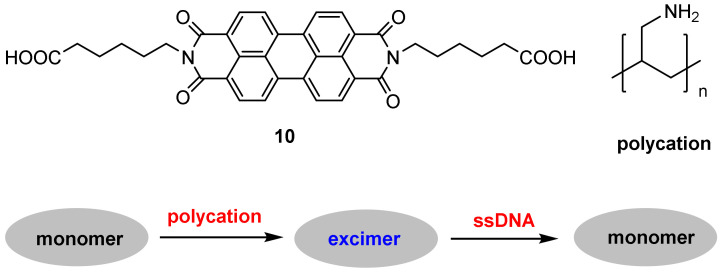
Chemical structures of probe **10** and polycation, and schematic representation for the detection of DNA MTase activity.

DNA methylation is a biochemical process in which a methyl group is added to cytosine or adenine bases of DNA nucleotides; this process is catalyzed by DNA methyltransferase (MTase) in the presence of S-adenosylmethionine (SAM). Thus, sensing DNA MTase activity and its inhibitor screening are of great importance for fundamental biochemical research, drug discovery and diagnosis of genetic diseases [67]. Wang and co-workers [68] designed a perylene excimer-based probe **10** for selective sensing of DNA MTase activity with the following working principle: A strong excimer fluorescence of probe **10** was induced by a cationic polymer (polycation) via electrostatic interactions. Upon addition of single-stranded DNA (an anionic polymer), strong electrostatic interactions between the polycation and the DNA weakened the binding of probe **10** to the polycation, resulting in the excimer fluorescence decreasing and monomer fluorescence increasing (Figure 23).

DNA adenine methylation (dam) MTase and the restriction endonuclease DpnI were chosen as the model MTase and endonuclease, respectively. When the 3′-OH terminus (3′-ddC) of a duplex DNA is removed, the DNA strands are not elongated by terminal deoxynucleotidyl transferase (TdT); in this case, the monomer fluorescence is detected. In the presence of MTase and endonuclease, the DNA is specifically methylated and cleaved into single-stranded fragments with new 3′-OH termini; in this case, the excimer fluorescence is produced.

**Figure 24:**
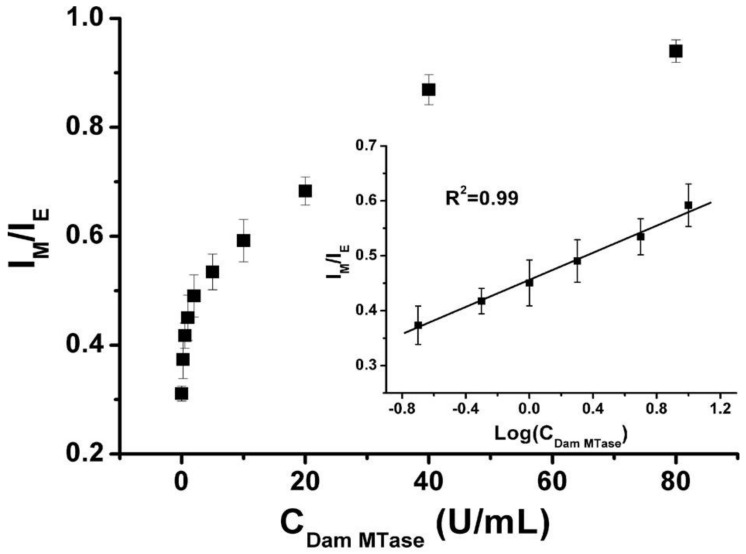
Plot of *F*_monomer_/*F*_excimer_ (I_M_/I_E_) value vs. *dam* MTase concentration (0, 0.2, 0.5, 1, 2, 2.5, 10, 20, 40, and 80 U/mL) [68].

Probe **10** exhibited high sensitivity and good selectivity. Maximum *F*_monomer_/*F*_excimer_ value was obtained after 2 h of enzymatic reaction with 80 U/mL *dam* MTase. The *F*_monomer_/*F*_excimer_ value increased gradually with the increase of *dam* MTase concentration, and the detection limit for *dam* MTase is calculated to be 0.2 U/mL (Figure 24). Control experiments demonstrated that other methyltransferases such as M.SssI MTase and HpaII MTase could not induce noticeable changes in the ratio of *F*_monomer_/*F*_excimer_.

**Figure 25:**
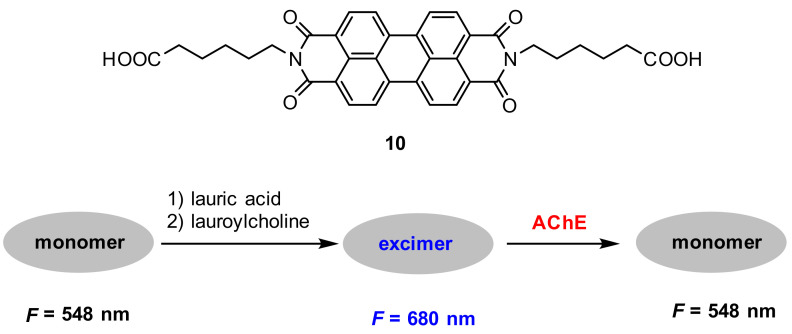
Chemical structure of probe **10** and schematic representation for the detection of AChE.

Acetylcholinesterase (AChE) plays an essential role in the catalysis of hydrolysis of the neurotransmitter acetylcholine [69]. The decrease in the level of acetylcholine boosts the assembly of amyloid β peptides into amyloid fibrils and generates Alzheimer’s disease (AD) [70,71]. Thus, it has great practical significance to create selective and sensitive probes for the detection of AChE. He and co-workers [72] employed **10** to construct a sensitive ratiometric fluorescence probe for the assay of AChE activity based on the monomer–excimer transition of probe **10** (Figure 25). The probe mechanism is as follows: probe **10** mainly existed in a monomer state in a buffer solution with two fluorescence peaks at 548 nm and 587 nm, respectively, aggregated to supramolecular assemblies by mixture with lauroylcholine (employed as the substrate of AChE) and lauric acid, resulting in a redshifted excimer fluorescence at 680 nm. Upon addition of AChE, the excimer transferred to the monomer by the hydrolysis of lauroylcholine to lauric acid and choline; as a result, the fluorescence change of the excimer–monomer transition was observed.

**Figure 26:**
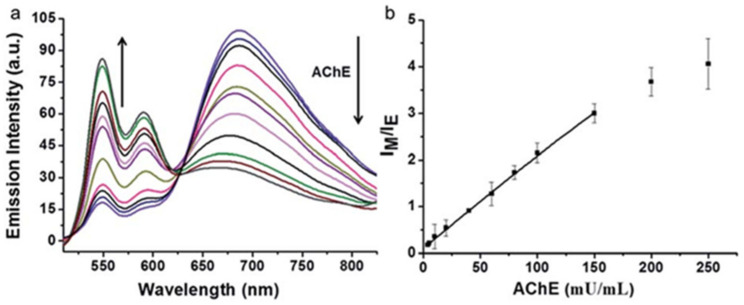
(**a**) Fluorescence response of probe **10** (10 μM) in MOPS (pH 7.4) solution with lauroylcholine (130 μM) and lauric acid (300 μM) to AChE (5–250 mU/mL). (**b**) Plot of *F*_monomer_/*F*_excimer_ (I_M_/I_E_) value vs. AChE concentration (5–250 mU/mL) [72].

The optical response of probe **10** to AChE is represented in Figure 26. In MOPS buffer solution (pH 7.5), the excimer fluorescence at 680 nm decreased gradually; meanwhile, the monomer fluorescence increased gradually with increased concentration of AChE (Figure 26a). The intensity ratios (*F*_monomer_/*F*_excimer_) of probe **10** plotted against the concentrations of AChE display a linear relationship at AChE concentration range from 5–150 mU/mL (*R*^2^ = 0.999). The limit of detection is calculated to be 5 mU/mL (Figure 26b).

**Figure 27:**
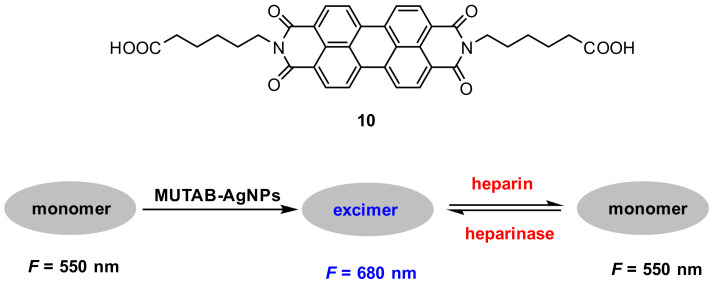
Chemical structure of probe **10** and schematic representation for sensing heparin and heparinase.

**Figure 28:**
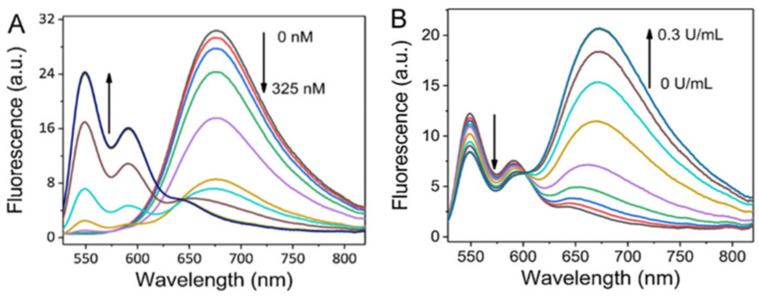
Fluorescence spectra of probe **10** in the presence of MUTAB-Ag NPs (29.4 ± 3.6 nm) with heparin (0–325 nM) (**A**) and with heparinase (0–0.3 U/mL) (**B**) [75].

Heparinase involves in various biological processes including angiogenesis, inflammation, tumor invasion and metastasis [73,74]. Li and co-workers [75] used probe **10** and cationic silver nanoparticles (Ag NPs) to construct a ratiometric fluorescence probe for sensing heparin and heparinase (Figure 27) with the following mechanism: A strong excimer fluorescence of probe **10** was induced by cationic Ag NPs which were stabilized by a ligand MUTAB via strong electrostatic interactions between them. These strong electrostatic interactions between the probe and MUTAB-Ag NPs were weakened when negative-charged heparin was adsorbed on the surface of MUTAB-Ag NPs, which blocked the formation of probe excimer, resulting in the decrease of *F*_excimer_/*F*_monomer_ (Figure 28A), whereas the degradation of heparin by heparinase facilitated the formation of the probe excimer, which resulted in the increase of *F*_excimer_/*F*_monomer_ (Figure 28B). The probe exhibited good sensitivity and excellent selectivity; the linear relationships for heparin and heparinase are in range from 0–75 nM and 0–0.25 U/mL, respectively.

**Figure 29:**
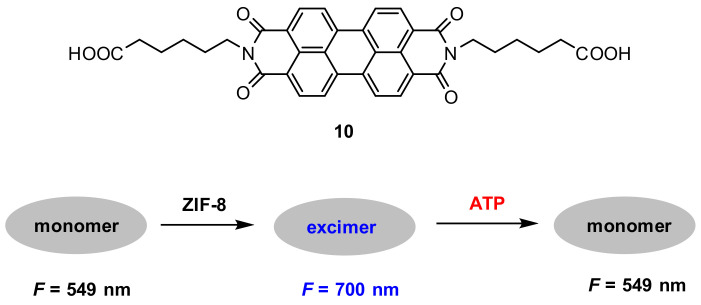
Schematic representation for the detection of ATP with probe **10@ZIF-8**.

Metal-organic framework (MOF) [76] is of great interest because of its potential applications in many fields due to its porous structure and tunable pore size. Zhou and co-workers [77] reported a ratiometric fluorescent probe for adenosine triphosphate (ATP) detection by encapsulating **10** into zeolitic imidazolate framework-8 (ZIF-8) nanocrystals. It was found that the formed **10@ZIF-8** exhibited significant excimer fluorescence at 700 nm; the excimer fluorescence was, however, reduced upon the addition of ATP due to strong binding between ATP and Zn^2+^. The strong binding between ATP and Zn^2+^ triggered the decomposition of **10@ZIF-8**, which resulted in the monomer fluorescence at 549 nm (Figure 29).

The fluorescence responses to ATP exhibited that the excimer fluorescence decreased gradually with the increase in the ATP concentration and a good linear relationship (*R*^2^ = 0.997) between the *F*_excimer_/*F*_monomer_ value and the ATP concentration (0–175 μM) was obtained. The detection limit for ATP is calculated to be 10 μM.

**Figure 30:**
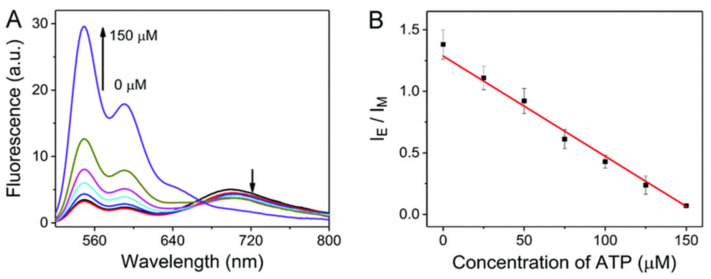
(**A**) Fluorescence spectra of probe **10@ZIF-8** with the addition of ATP (0–150 μM) in cell lysates (5%). (**B**) Corresponding *F*_excimer_/*F*_monomer_ (I_E_/I_M_) value based calibration curve for ATP (0–150 μM) in cell lysates (5%) [77].

The application of the probe to quantify ATP in cell lysates was conducted. Cell lysates (5%) were spiked with ATP in different concentrations. The value of *F*_excimer_/*F*_monomer_ was linearly related (*R*^2^ = 0.992) to the ATP concentration in the range from 0–150 μM (Figure 30). The concentration of ATP in the cell lysates was calculated to be 3.1 mM.

### 3.3. Benzothiazole-Based Probes

**Figure 31:**
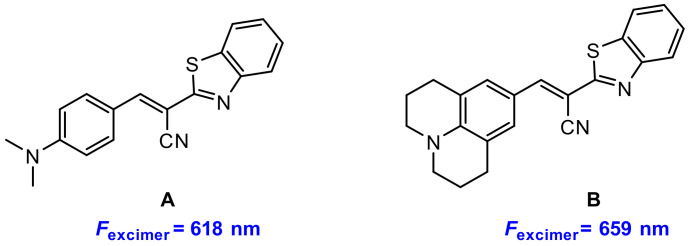
Chemical structures of benzothiazole derivatives **2** and **3**.

Recently, Kim’s group and Bouffard’s group [78] reported two benzothiazole-based excimers with red fluorescence and large Stokes shifts. They synthesized two benzothiazole derivatives **A** and **B** (Figure 31), and found that both **A** and **B** exhibited aggregation in aqueous solution (acetonitrile–water = 1:9, *v/v*) resulting in excimer fluorescence at 618 nm and 659 nm, respectively.

**Figure 32:**
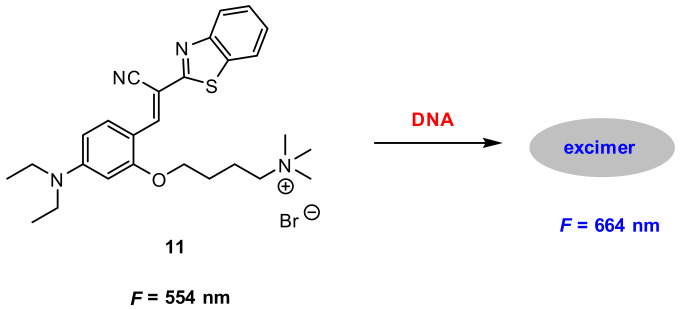
Chemical structure of **11** and its response to DNA.

Based on the above findings, they developed a benzothiazole derivative-based dye **11** (Figure 32) for the label-free detection of DNA. Probe **11** displayed a very weak fluorescence at 544 nm in buffer solution HEPES (pH 7.4). Upon addition of double-stranded (ds) calf-thymus DNA (dsDNA), a strong excimer fluorescence at 664 nm was detected. The fluorescence intensity of *F*/*F*_o_ at 664 nm increased linearly with the increase of dsDNA concentration until saturation was reached. In this case, the concentration of dsDNA was approximately 50 pM (0.43 ng/µL) and the fluorescence was enhanced 21-fold.

**Figure 33:**
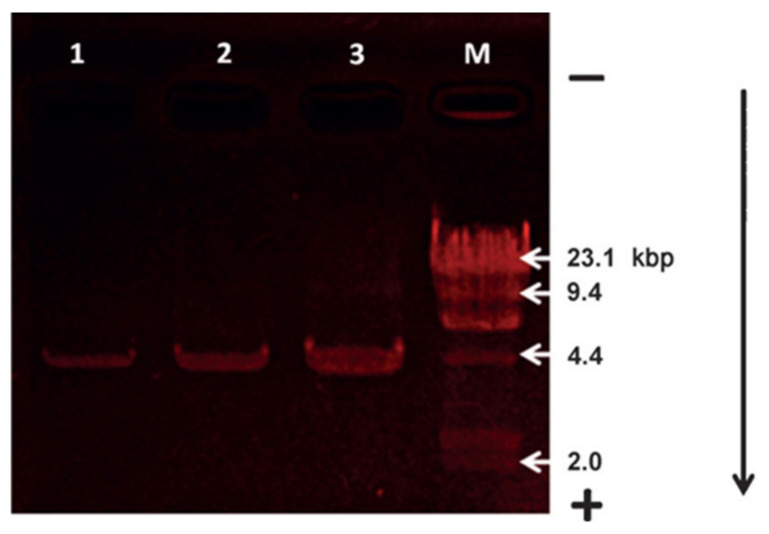
Image of agarose gel electrophoresis assays of plasmid DNA (4.1 kbp). Lanes 1, 2, and 3 were loaded with 64 mg, 128 mg, and 320 mg of DNA, respectively. Lane M: DNA molecular-weight marker. The gel was poststained by probe **11** (50 µM) for 60 min. The arrow indicates the direction of DNA migration [78].

Probe **11** can also be used as a staining agent for the gel electrophoresis of DNA (Figure 33). Plasmid DNA was separated on agarose gel before staining with a solution of **4** and rinsing with water. Red emission bands indicating electrophoretic DNA migration were observed.

**Figure 34:**
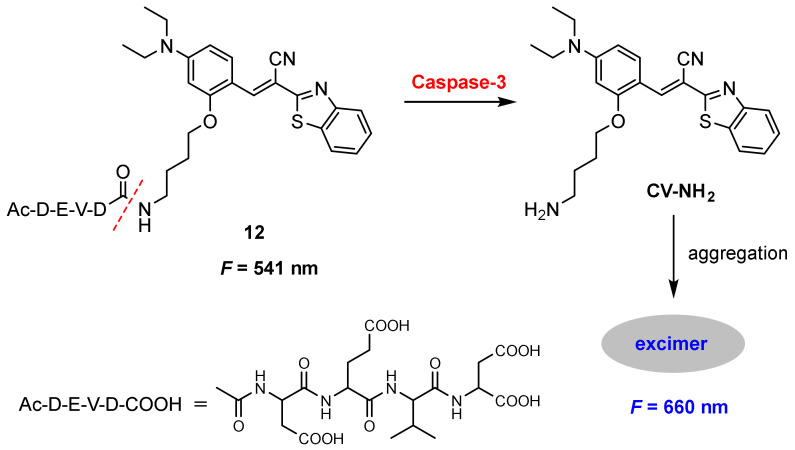
Chemical structure of probe **12** and its response to caspase-3.

Caspases play essential roles in the initiation and execution of the apoptotic pathway as well as in inflammation. Accurate assessment of caspase activity can provide valuable information for biomedical research and drug development [79,80,81]. Kim and co-workers [82] developed a benzothiazole-based excimer fluorescent probe for the detection of caspase-3. Probe **12** was constructed by a fluorophore (**CV-NH_2_**) and a caspase-3 recognition peptide (**DEVD**) through an amide bond. The probe displayed weak fluorescence in aqueous media. The cleavage of **DEVD** peptide by caspase-3 produced the **CV-NH_2_** residue, which aggregated in aqueous solution and resulted in excimer fluorescence (Figure 34).

The response to caspase-3 was explored by monitoring the fluorescence change of **Ac-DEVD-NH-CV**. The red fluorescence at 660 nm increased upon the addition of caspase-3. The probe exhibited high selectivity for the detection of caspase-3 and the quantitative analysis of caspase-3 with probe **12** was obtained by the ratio of *F*_660_/*F*_541_. The ratio (*F*_660_/*F*_541_) showed 95-fold enhancement incubation with caspase-3 from 0 to 150 ng/mL. A linear relationship in the range from 0–3 ng/mL (*R*^2^ = 0.9938) was obtained and the limit of detection for caspase-3 was determined to be 5.1 pg/mL (0.17 pM).

**Figure 35:**
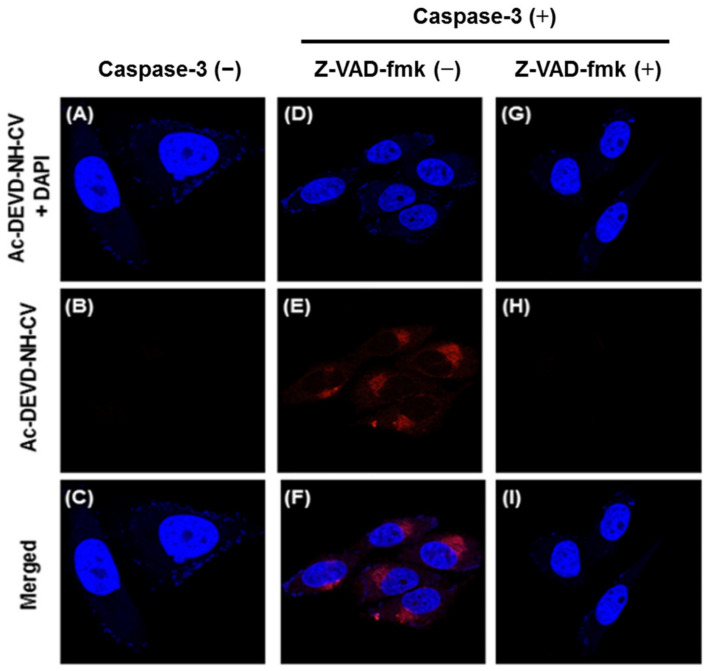
Fluorescent images of HeLa cells incubated with probe **12** (10 μM) under different conditions. The cells were incubated with probe **12** for 2 h before (**A**–**C**) and after treatment with caspase-3 in the absence (**D**–**F**) or in the presence (**G**–**I**) of caspase-3 inhibitor Z-VAD-fmk (50 nM) for 2 h. The cells were co-stained with the blue fluorescent DNA binding dye DAPI. Top: E_x_ = 405 nm, E_m_ = 410–476 nm; middle: E_x_ = 488 nm, E_m_ = 550–696 nm; bottom: merged images [82].

In vitro imaging of caspase-3 with probe **12** was performed in HeLa living cells. A protein delivery system loaded with 150 ng/mL caspase-3 onto AuNP–His–Apt-composite, and was used to deliver caspase-3 into cells. A red fluorescence was observed when the **12**-loaded cells treated with AuNP–His–Apt–caspase-3 complex [83] (Figure 35E). By contrast, no fluorescence was observed without the complex (Figure 35B). Furthermore, significantly decreased fluorescence was detected when the **12**-loaded cells were pretreated with the inhibitor Z-VAD-fmk [84] before incubation with the complex (Figure 35H). Results showed that the probe can permeate efficiently into the cells and be activated by caspase-3. Co-staining with the DNA indicated that the probe accumulated in the cytosolic compartment of the cells.

## 4. Conclusions and Outlook

In the past decade, considerable progress has been made in exploring excimer-based fluorescent probes for the detection of bioactive molecules and biological process due to convenient availability, high sensitivity and superior bioimaging capability. Many outstanding excimer-based fluorescent probes [85,86,87,88,89,90,91] are not involved herein due to the length of the article. In this review, fluorescent probes based on organic small-molecules are summarized (Table 1) including design strategies, reaction mechanism and applications to biological detection or imaging.

Although great progress has been made, however, some problems remain for practical applications: most pyrene-based probes suffer from short excitation and emission wavelength which limit their applications in biological system. Perylene-based probes exhibit poor solubility in both organic solvent and aqueous solution. In addition, the types of molecules with excimer emission are not rich enough, and more novel molecular structures with excimer emission need to be found and studied.

From a practical point of view, the new fluorescent probes based on excimer emission will be further explored in the future and several characteristics should be considered. First, highly sensitive, selective and accurate detection is required especially in early-stage diagnoses, which can reduce the mortality rate. Second, for the in vivo detection and imaging, NIR or multi-photon fluorescent probes are desired because of their low background signal and deep penetration depth. Third, quantitative detection is needed since bioactive molecules exhibit different levels of activity at different stages of disease development. In addition, solubility, stability, cell penetrability and cytotoxicity are important parameters in practical applications, which also need to be considered in the design of probes.

## Figures and Tables

**Table 1:** Highlights of fluorescent probes included in this review for biological applications.

Probe	^a^ MA	^b^ λ_em_ (nm)	^c^ Δλ (nm)	Analyte	^d^ LOD	^f^ Ref.
1	A	480	120	protease MMP-7	^e^ ND	[33]
2	A	480	130	diphosphorylated protein	0.6 µM	[35]
3	A	485	120	lgE	1.6 nM	[36]
4	B	482	122	OCl^−^	0.2 µM	[37]
5	B	470	120	OCl^−^	0.35 µM	[40]
6	B	459	114	intracellular pH	-	[44]
7	A	607	107	cell imaging	-	[59]
8	A	670	150	cell imaging	-	[63]
9	A	552	82	fingerprint	-	[66]
10	B	680	180	DNA MTase	0.2 U/mL	[68]
10	B	680	180	acetylcholinesterase	5 mU/mL	[72]
10	B	680	180	heparin and heparinase	ND	[75]
10	B	700	200	ATP	10 µM	[77]
11	B	664	198	dsDNA	ND	[78]
12	B	660	189	caspase-3	0.17 pM	[82]

^a^ MA, mechanism; A, off-on fluorescence; B, ratiometric fluorescence. ^b^ λ_em_, maximum emission wavelength of excimer under physiological conditions. ^c^ Δλ, Stoke’s shift (Δλ = λ_em_ − λ_abs_) of excimer under physiological conditions. ^d^ LOD, the limit of detection. ^e^ NR, no report. ^f^ Ref., reference.

## Data Availability

Not applicable.
